# Her2 positivity and race predict higher mastectomy rates: a SEER database analysis

**DOI:** 10.1186/s40064-015-1538-x

**Published:** 2015-11-24

**Authors:** Theresa L. Schwartz, Jula Veerapong, Leslie Hinyard

**Affiliations:** Department of Surgery, Saint Louis University, 3635 Vista Avenue, DT, 3rd Floor, Saint Louis, MO 63110 USA; Saint Louis University Center for Outcomes Research, 3545 Lafayette Ave., Salus Center, Room 409, Saint Louis, MO 63104 USA

**Keywords:** Breast cancer, HER2, SEER, Receptors disparities

## Abstract

Given the difference in incidence of biologic subtype of breast cancer between black and white women, and the potential disparity in type of surgery in black and white women presenting with early stage breast cancer, this study aimed to examine the odds of mastectomy compared to lumpectomy by molecular subtype in black and white women with size T1 and T2 invasive breast cancer. Using the SEER database, breast operation choice for women over the age of 15 with T1 or T2 tumors between 2010 and 2012 were examined. Tumors were categorized according to the Breast Subtype variable in the SEER database and data were stratified by tumor size and race. Bivariate comparisons and logistic regression models adjusted for age were used. In women with T1 or T2 tumors, mastectomy rates were higher in women with Her2 positive tumors than in those with Her2 negative tumors. When Her2 results are the same among comparison groups, those women with HR positive tumors were less likely to undergo a mastectomy than those with HR negative tumors. In T1 tumors, the magnitude of the association was larger for white women than women of other races. Results suggest there are differences in surgical decision making based on breast cancer subtype in women with T1 or T2 tumors and that race may play a role for size T1 tumors. The strong association between Her2 positive tumors and higher mastectomy rates warrants further investigation.

## Background

Breast cancer is a heterogeneous malignancy which has been classified by gene-expression profiling into distinct molecular subtypes that provide important prognostic and predictive information (Perou et al. 2000; Sorlie et al. 2001). Estrogen receptor (ER), progesterone receptor (PR) and human epidermal growth factor receptor-2 (Her2) represent the elements that define these breast cancer subtypes and are routinely obtained to guide systemic therapy decision making. Marked differences have been noted in long-term breast cancer related outcomes according to disease subtype, including significantly worse recurrence rates and overall survival in those patients with Her2 positive tumors and tumors that lack expression of ER, PR and Her2 (triple negative) (Sorlie et al. 2001, 2003; Carey et al. 2006; Slamon et al. 1987; Ravidn and Chamness 1995). Equivalent disease-free and overall survival for breast-conserving surgery—lumpectomy followed by radiotherapy—and mastectomy has been well described in multiple, large, randomized controlled trials (Fisher et al. 2002; Veronesi et al. 2002). These trials were performed prior to the implementation of biological subtype differentiation; therefore, no differences in outcome based on type of surgical treatment received according to subtype can be ascertained from these studies. In the modern era, there have been discordant findings regarding locoregional recurrence rates between subtypes. While it would be logical to question the use of breast-conserving surgery in those patients with more aggressive tumor biology in the triple negative or Her2 positive cohorts, multiple studies have demonstrated no significant difference in LRR related to the subtypes (Freedman et al. 2009; Haffty et al. 2006; Peterson et al. 2014; Gangi et al. 2014). There is no current evidence to suggest that the surgical management of early stage breast cancer should differ based on tumor subtype.

In the United States, there are documented differences in the racial distribution of biologic subtype, type of surgery, and treatment outcomes for breast cancer. White women present with the highest incidence of ER/PR positive/Her2 negative tumors while black women have the highest incidence of triple negative tumors (Amend et al. 2006; Howlader et al. 2014). Five year survival is lower for black women compared to white women across subtypes (Chen et al. 2014), and there is a demonstrated difference in disease free survival by race and molecular subtype (Sparano et al. 2012). There is some evidence that black women are more likely to undergo mastectomy compared to white women; however, it is unclear if this is due to later stage at time of diagnosis (Sparano et al. 2012; Dookeran et al. 2015).

A recent analysis of SEER data found that women with Her2 positive disease, regardless of hormone receptor positivity, have higher odds of mastectomy compared to those who are Her2 negative/HR positive. Women with triple negative breast cancer also had higher odds of mastectomy compared to the Her2 negative/HR positive women (Lizarraga et al. 2010). The study controlled for race and tumor size, but did not examine the potential interaction between race and biologic subtype. Given the difference in incidence of biologic subtype between black and white women, as well as the potential disparity in type of surgery in black and white women presenting with early stage breast cancer, this study aimed to examine the odds of mastectomy compared to lumpectomy by molecular subtype in black and white women with size T1 and T2 invasive breast cancer.

## Methods

This study used the National Cancer Institute’s Surveillance, Epidemiology and End Results (SEER) 18 regions research database. Women between the ages of 15 and 85+ years diagnosed with T1 or T2 invasive breast cancer between the years 2010 and 2012 were included in the sample. During these years in the SEER database, Her2 status was routinely recorded for all invasive breast cancers and anti-Her2 therapy was considered standard of care for patients with Her2 positive disease. For women with multiple tumors over their lifespan, only the first primary tumor was included in the analysis. Women whose first primary tumor was not invasive breast cancer, with a primary tumor diagnosed prior to 2010 and those without complete information on hormone receptor (HR)—which includes ER and PR—and Her2 status were excluded.

Tumors were categorized as the following: Her2+/HR+, Her2+/HR−, Her2−/HR+ and Her2−/HR− using the breast subtype variable available in the SEER database (Howlader et al. 2014). Data were stratified by tumor size (T1 or T2) and race. Bivariate comparisons were made using Chi square (χ^2^) and logistic regression models adjusted for age were used to determine the odds of mastectomy by breast cancer molecular subtype. All analyses were conducted using SAS 9.3 (Cary, NC).

## Results

The final sample included 112,963 women who were diagnosed with a T1 or T2 invasive breast cancer between 2010 and 2012. Demographic information for the sample stratified by surgery type is outlined in Table 1. Overall, women who underwent a mastectomy were more likely to be younger, have a T2 tumor and be of a race other than white.

**Table 1:** Demographic characteristics stratified by surgery type (N = 112,963)

	Lumpectomy N (%)	Mastectomy N (%)	p
Age			
15–49	11,532 (17)	13,011 (29)	<0.0001
50–85+	56,607 (83)	31,813 (71)	
Race			
White	55,666 (82)	35,341 (79)	<0.0001
Black	6865 (10)	4,683 (10)	
Other	5,608 (8)	4,800 (11)	
Tumor size			
T1	51,079 (75)	24,200 (54)	<0.0001
T2	17,060 (25)	20,624 (46)	
Biologic subtype			
HR+/Her2+	5,775 (8)	5,294 (12)	<0.0001
HR−/Her2+	2,008 (3)	2,500 (6)	
HR+/Her2-	53,214 (78)	31,604 (70)	
Triple negative	7,142 (10)	5,426 (12)	

Results of the stratified regression models are reported in Table 2. For women with T1 tumors, there was a statistically significant interaction between tumor subtype and race (χ^2^ = 21.2, p = 0.002). For this reason, in women with T1 tumors, the results are stratified by race. There was not a statistically significant interaction between tumor subtype and race in T2 tumors (χ^2^ = 8.9, p = 0.18) and the results are presented for all races combined.

**Table 2:** Age-adjusted odds ratios of mastectomy to lumpectomy between breast cancer biologic subtypes for T1 tumors stratified by race and T2 tumors

T1	White	Black	Other	T2	OR (95 % CI)
	OR (95 % CI)	OR (95 % CI)	OR (95 % CI)		
Her2+/HR+ vs Her2+/HR−	0.63 (0.57, 0.70)	0.86 (0.65, 1.13)	0.61 (0.47, 0.80)	Her2+/HR+ vs Her2+/HR−	0.66 (0.60, 0.72)
Her2+/HR+ vs Her2−/HR+	1.44 (1.36, 1.53)	1.30 (1.10, 1.52)	1.33 (1.12, 1.57)	Her2+/HR+ vs Her2−/HR+	1.42 (1.34, 1.50)
Her2+/HR+ vs triple negative	1.20 (1.10, 1.30)	1.42 (1.16, 1.72)	1.12 (0.88, 1.44)	Her2+/HR+ vs triple negative	1.22 (1.14, 1.32)
Her2+/HR− vs Her2−/HR+	2.28 (2.08, 2.50)	1.51 (1.19, 1.91)	2.17 (1.72, 2.74)	Her2+/HR− vs Her2−/HR+	2.16 (1.99, 2.34)
Her2+/HR− vs triple negative	1.89 (1.69, 2.10)	1.65 (1.27, 2.14)	1.84 (1.37, 2.47)	Her2+/HR− vs triple negative	1.87 (1.70, 2.05)
Her2−/HR+ vs triple negative	0.83 (0.78, 0.88)	1.09 (0.95, 1.26)	0.85 (0.69, 1.04)	Her2−/HR+ vs triple negative	0.86 (0.82, 0.91)

### T1 tumors

For white women with T1 tumors, the odds of mastectomy differed based on biologic subtype. When white women with the same Her2 status were compared according to differing HR status, those with HR negative tumors were more likely to undergo a mastectomy than those with HR positive tumors (Her2+/HR+ vs Her2+/HR− OR = 0.63, 95 % CI 0.57, 0.70; Her2−/HR+ vs Her2−/HR− OR = 0.83, 95 % CI 0.78, 0.88). When the Her2 status was different between comparison groups, white women with Her2+ tumors were more likely to undergo a mastectomy, regardless of HR status, although the strength of the association varied. The largest association was seen with Her2+/HR− vs Her2−/HR+ (OR = 2.28, 95 % CI 2.08, 2.50), followed by Her2+/HR− vs Her2−/HR− (OR 1.20, 95 % CI 1.10, 1.30).

Black women with T1 tumors had a similar, though not identical, pattern. However, the magnitude of associations is smaller between most biologic subtypes compared to white women and women of other races. In black women with T1 tumors, when Her2 status is the same between comparison groups, there is no statistically significant difference in odds of mastectomy, regardless of HR status (Her2+/HR+ vs Her2+/HR− OR = 0.86, 95 % CI 0.65, 1.13; Her2−/HR+ vs Her2−/HR− OR 1.09, 95 % CI 0.95, 1.26). When comparing those with differing Her2 status, black women with Her2+ tumors were more likely to undergo a mastectomy (Her2+/HR− vs Her2−/HR− OR = 1.65, 95 % CI 1.27, 2.14; Her2+/HR− vs Her2−/HR+ OR 1.51, 95 % CI 1.19, 1.91; Her2+/HR+ vs Her2−/HR− OR = 1.42, 95 % CI 1.16, 1.72; Her2+/HR+ vs Her2−/HR+ OR = 1.30, 95 % CI 1.10, 1.52).

When comparing women of other races with T1 tumors and positive Her2 status, those with HR negative tumors were more likely to undergo a mastectomy than those with HR positive tumors (Her2+/HR+ vs Her2+/HR− OR = 0.61, 95 % CI 0.47, 0.80). However, when Her2 status is negative, there is no statistically significant difference in odds of mastectomy by differing HR status (Her2−/HR+ vs Her2−/HR− OR 0.85, 95 % CI 0.69, 1.04). When comparing those with differing Her2 status, women with Her2 positive tumors were more likely to undergo a mastectomy (Her2+/HR− vs Her2−/HR− OR 1.84, 95 % CI 1.37, 2.47; Her2+/HR− vs Her2−/HR+ OR 2.17, 95 % CI 1.72, 2.74; Her2+/HR+ vs Her2−/HR+ OR 1.33, 95 % CI 1.12, 1.57), with the exception of the Her2+/HR+ vs Her2−/HR− comparison, which was not statistically significant (OR = 1.12, 95 % CI 0.88, 1.44).

### T2 tumors

There was no statistically significant interaction between tumor subtype and race in T2 tumors. For all races combined, when comparing groups with the same Her2 status but differing HR status, women with HR positive tumors were less likely to undergo a mastectomy than those with HR negative tumors (Her2+/HR+ vs Her2+/HR− OR 0.66, 95 % CI 0.60, 0.72; Her2−/HR+ vs Her2−/HR− OR = 0.86, 95 % CI 0.82, 0.91). When comparing groups with differing Her2 status, women with Her2 positive tumors were more likely to undergo a mastectomy than those with Her2 negative tumors, although the magnitude varied. The largest association was seen between Her2+/HR− vs Her2−/HR+ tumors (OR = 2.16, 95 % CI 1.99, 2.34), followed by Her2+/HR− vs Her2−/HR− (OR 1.87, 95 % CI 1.70, 2.05), Her2+/HR+ vs Her2−/HR+ (OR = 1.42, 95 % CI 1.34, 1.50) and Her2+/HR+ vs Her2−/HR− (OR = 1.22, 95 % CI 1.14, 1.32).

## Discussion

The results of this study suggest that HR status and Her2 status may influence mastectomy rates in women with T1 and T2 tumors who were diagnosed between 2010 and 2012. These women were diagnosed after the use of anti-Her2 therapy became standard of care in women with Her2 positive disease. In T1 tumors, the odds ratios of mastectomy to lumpectomy black women are smaller than those for white women and women of other races. The direction of the relationship is the same, however, the effect sizes for the odds of mastectomy are different with the largest discrepancy being for the comparison of Her2+/HR− and Her2−/HR+ tumors. Even with the differences across races in T1 tumors, general patterns can be seen in surgical decision-making across races and tumor size. In general, when Her2 status is the same between comparison groups, positive HR status is associated with a reduced odds of undergoing a mastectomy. More interestingly, when Her2 status is different between comparison groups, Her2 positivity is associated with an increased odds of undergoing a mastectomy.

There is limited, and conflicting, data on the relationship between Her2 status and the risk of locoregional recurrence. Nguyen and colleagues previously reported a higher rate of local recurrence in Her2 positive patients compared to HR positive/Her2 negative patients (adjusted hazard ratio = 9.2, 95 % CI 1.6, 51; p = 0.012); however, none of the patients in this 2008 study received anti-Her2 therapy, which has become standard of care in patients with Her2 positive disease (Nguyen et al. 2008). It has been well-established that the use of adjuvant anti-Her2 therapy in the treatment of Her2 positive tumors reduces the risk of both local recurrence as well as distant metastasis (Piccart-Gebhart et al. 2005; Romond et al. 2005). Therefore, it is unknown if a higher recurrence rate would be realized if these women had received modern anti-Her2 regimens. Without definitive evidence of higher locoregional or distant metastasis in women with early stage breast cancer, the reasoning behind higher mastectomy rates in Her2 positive disease remains unclear.

There are limitations to this study. First, we excluded women with missing HR or Her2 status. The overall sample had 2.6 % missing ER status, 3.1 % missing PR status and 7.1 % missing Her2 status. The missing data were not randomly distributed across age, race, SEER registry or tumor size. It is unclear how much the missing data may bias results or the direction of any potential bias and may lead to a differential under-or over-estimation of the association between biologic subtypes and mastectomy rates. Second, neither multifocality nor multicentricity is a documented variable in the SEER database. Using T-stage only, without knowledge of the possibility of multifocality or multicentricity, may grossly underestimate the extent of disease within the breast. This may have been a confounding factor in a woman’s surgical decision-making process. Third, there is no information in the SEER database regarding family history of malignancies. Although this should be evenly distributed across all molecular subtypes, it is impossible to know if a significant family history of cancer may have contributed to the decision to proceed with a mastectomy.

The phenomenon of increasing mastectomy rates in early stage breast cancer has been well described. In an analysis of the National Cancer Database, Kummerow et al. reported a 37.8 % mastectomy rate in 2011 in women with early stage breast cancer (2015). These results are consistent with other long term studies investigating the use mastectomy over breast-conserving surgery (Dragun et al. 2013; McGuire et al. 2009). With this trend in mind, it is even more imperative that differences in mastectomy rates be continuously investigated. There are no guidelines or evidence based recommendations suggesting that surgical decision-making should be based on tumor biology. The underlying knowledge that Her2 positive disease tends to be a more aggressive phenotype may lead both patients and physicians to falsely believe breast-conserving surgery to be unsafe, despite no evidence to suggest that this is the case. The strong association between Her2 positive disease and mastectomy rates described in this study suggests that further investigation is needed into how information about the diagnosed cancer and the possible surgical options are presented to a patient. Identifying the factors involved in the surgical decision-making process is necessary to ensure all information provided to patients is evidence based, rather than perception based. A better understanding of the delivery of information may shed some light on the observed differences in mastectomy rates based on tumor subtype.
